# Notices of the Lunatic Asylums of Paris, &c.

**Published:** 1845-07

**Authors:** John Conolly

**Affiliations:** Fellow of the Royal College of Physicians, Physician to the County Lunatic Asylum at Hanwell


*Notices of the Lunatic Asylums of Paris, &c. In a letter addressed to* John Forbes, M. D. F. R. S. *By* John Conolly, M. D. *Fellow of the Royal College of Physicians, Physician to the County Lunatic Asylum at Hanwell.*

CHARENTON.

The French physicians begin their labours at an early hour; and at eight o'clock on a fine morning in November I had the pleasureof accompanying M. Foville to the celebrated asylum of Charenton, situated a few miles east of Paris, on the elevated bank of the Marne. Sixteen years before, I had visited this asylum, of which Esquirol was then the chief physician. He succeeded the eminent Royer-Collard. The appointment was offered to Pinel in 1797, when Charenton was opened as an asylum for the insane; but he could not make up his mind, Esquirol says, to leave his poor patients and his pupils at the Salpêtrière. M. Royer-Collard was succeeded by Esquirol, at whose death M. Foville was appointed. Thus to revisit the same place, after even the short interval of a few years, is to become more sensible of the gradual changes which time is always effecting; of the removal of individuals, and the onward progress of all those works which successions of individuals are ordained to accomplish. Esquirol has been removed from the scene of his great and useful exertions; but the asylum to which fourteen of his latest and most experienced years were devoted is undergoing that entire renewal for which he so anxiously wished. Old and inconvenient structures are disappearing, and new and elegant buildings are in progress which almost assume the character of grandeur. Whilst these alterations afford opportunities for numerous improvements, both in the construction of the asylum and the management of the patients, strikingly conspicuous at present, when a part of the old building still remains, it is questionable whether so great an expenditure as is now incurred should have been applied to a building in such a site; the surrounding ground, although the asylum is on an agreeable eminence, being rocky, precipitous, and unfavorable to the formation of airing-courts, or obtaining space and productive soil for gardens affording recreation, or fields furnishing employment. These natural inconveniences are alluded to in M. Esquirol's description of the new part of the asylum on the female side, completed in his time, and which have still very great advantages over the older parts of the building. The attempt to adapt the buildings of old religious houses, fortresses and prisons, to the purpose of an asylum, is usually unsuccessful. In the case of Charenton, formerly a religious house, this was from the first detrimental to the old plan, and an adherence to the same locality seems to be almost equally so to the new.

The disposition of the older parts of the asylum of Charenton is so extremely irregular as to defy intelligible verbal description; that of the new, but yet unfinished part, appeared to have the general character of several detached quadrangles or courts, built on each side of a central church of Greek architecture. The material is a beautiful stone. On three sides of each of these courts or quadrangles are handsome buildings of two floors, and on the third side, to the south, a lofty iron railing, through which is obtained a fine and extensive prospect. This noble institution is chiefly destined to the reception of patients of the educated classes of society, or of the middle classes; literary and scientific men, artists, merchants, ecclesiastics, officers of the army and navy, persons in public offices, or their wives or children, when affected with insanity. They are received at different rates of payment, principally in three classes; and some, nominated by the Minister of the Interior, are admitted gratuitously. The annual payments range from 720 to 1300 francs per annum. Some patients are placed in the asylum at reduced rates of payment, also on the recommendation of the Minister of the Interior, and pay only one half or one third of the usual charge. For these an annual sum is paid out of the public fund, by the government. Fourteen beds are reserved for the pauper lunatics of the canton. At present the number of patients is about 450; but with the new buildings a considerable enlargement is contemplated, a circumstance certainly to be regretted. The general adaptation of the building is suitable to those intended to inhabit it; but although its character, taken altogether, and its fine situation are striking, the appearance of the square courts affects the spectator unfavorably; they are extremely clean, and command fine prospects, but are not sufficiently spacious entirely to remove the idea of a prison, derived from the elevated and apparently inaccessible situation of the asylum. I found such to be the impression made on the Parisians, and it is, perhaps, disadvantageous to the institution. All the interior arrangements are, however, most unobjectionable. The single rooms for patients, of which there are many, are of good size, excellent height, have large cheerful windows, and are comfortably furnished. The number of large dormitories appeared to me to be very small in proportion to the single rooms, at least in the new building; and I think M. Foville scarcely participates in the general partiality for them which prevails in France. In different parts of the asylum, the furniture and arrangements of the rooms are of course various; but there is great comfort throughout all those of recent construction. The apartments for several of the patients have a small room attached to them for an attendant; and I was glad to see that these were neither dark nor inconvenient, as is too generally the case in the older asylums, in which the attendant received no consideration. The truth slowly makes its way that good attendants make good patients; and that the way to make good attendants is to choose respectable persons and to make them comfortable. The importance of this very simple truth in asylums is immense. In all the French asylums, the beds are worthy of admiration. At Charenton almost all the bedsteads are of iron. The diet, varied according to the habits of the patients, is abundant and excellent. Patients of a certain rank dine with the director, or in their own apartments. All the patients are neatly and becomingly dressed in clothes provided by their friends. The military and naval patients receive clothing from the establishment. Great order and exactness characterizes all the arrangements relative to these matters; but they are too many for detail: although, like everything in this well-governed institution, deserving of the utmost attention from the directors of similar establishments.

As many or most of the female patients at Charenton are gentlewomen, and many of the men are old officers, or persons accustomed to the various impressions of social life in classes enjoying a certain degree of comfort, a great variety of resources becomes not only available in their treatment, but necessary. Among the women, all the kinds of needlework form a large part of their daily occupation; but reading, and drawing, and music contribute to diversify their time. Gardens and terraces of considerable variety and beauty offer them inducements to take exercise out of doors. Among the men, reading and writing are the most prevalent modes of employing themselves; music and billiards are among their amusements. Many, as in all asylums, prefer walking about to any occupation. Some of the most tranquil women were in large and comfortable day-rooms, in which there were several tables and chairs; but they seem, as usual with the insane, to lead an isolated life; many living much in their rooms, and few appearing to associate intimately with the rest. Most of the men seemed to live much alone, and the apartments of some had the air of a quiet study or bureau, in which our visit created a kind of temporary interruption. Patients of the higher classes fall most easily into a monotonous course of life when insane; and the most active ingenuity of a director can scarcely redeem them from it. In one room, used as a school-room, a patient was acting as schoolmaster or lecturer to the rest; demonstrating the situation of various countries on a large map, which he did quite correctly, although with occasional hesitation; and it was interesting to notice his successful struggles now and then to deliver himself from some difficulty of expression, or some temporary forgetfulness, or some transient confusion in his thoughts.

Every evening some of the patients, named by the physician, and usually including the convalescents, are assembled in a kind of a common room, or *salon*, for the sake of society and social enjoyments; some of the officers of the asylum being always present. Something of this kind is much wanting in almost all our large English asylums. The opportunities and advantages of such recreation are too rare; for their effects when enjoyed are very remarkable and evidently salutary. Constant work and formal discipline, transferred from prisons and work-houses, cast a gloom over many patients and create much avoidable discontent. It seems to be forgotten that most of the minds to be dealt with in asylums are *spoiled* for continuous application, efficient labour, or invariable order and propriety; and scarcely less forgotten that many require to be roused into a capacity for exertion, rather than to be stimulated to exert themselves regularly. Many young patients, especially, are to be noticed in the wards of asylums, of whom the history is, that after a short attack of maniacal excitement, they have become listless, apathetic, and indolent; but these patients understand everything that is said to them, and many of them are neither affected with delusions or bewilderment of mind. The brain seems to have lost much of its energy; and their tendency is towards total imbecility. Such patients are unwilling, generally indeed unable to work; and therefore they fall into disfavour with all but medical observers. Under an austere system they perish; falling gradually into fatuity or dimentia. They require encouragement; and for them cheerful recreation, frequent amusements, various exercise, mingled often with kind remonstrances, are remedial. They are more benefitted by drawing, music, and children's games, than by medicine. Among patients of a higher class, as at Charenton, social appliances become of wider applicability. In these attempts, however, as in the whole treatment of insanity, a moderation must ever be preserved. Lunatics bear no violent impressions with benefit, or even with impunity. The appliances should be very frequent; but never long continued, and never excessively exciting.

M. Foville's writings on Insanity and on the Nervous System are so well known in England, that it is scarcely necessary to say that he is a physician of great powers of observation and reflection, anxious for every improvement, but careful of hasty innovation, and habitually exercising an accurate judgment on whatever falls within the department of medicine in which he is now so experienced. The annual Reports of Charenton, presented to the Minister of the Interior, are not published ; a circumstance which probably deprives the public of some of those fresh and valuable impressions suggested in the constant attendance of a physician in an institution presenting a large and interesting field for observation; and the value of which might be gathered even from the casual remarks made by this accomplished physician in the course of the long morning visit in which I was permitted to accompany him. His general treatment of his patients appeared to me to be essentially the same as that adopted by English practitioners in mental disorders; consisting of means of regulating the functions of the digestive organs and the skin, equalizing the circulation, allaying nervous disturbance, and soliciting a renewal of natural cerebral exercise. In his intercourse with his patients he is extremely mild, composed, and judicious ; never attempting too much at once, or endeavouring to force powers that require considerate direction rather than stimulus; and evidently doing only what he considers to be really useful, and nothing from ostentation. Thus M. Foville does not assume the office of lecturer as he goes his rounds, or make the least attempt to convince the visitor that he is enjoying a great privilege. But the whole demeanor of a sensible physician when he goes through the wards of an asylum is instructive ; everything has its meaning and its object. Vivacious visitors too often forget this ; talk too much, suggest too hastily, discuss too earnestly, and observe too little ; oblivious that the physician is actually applying various proportions of mental remedy at every step. I need scarcely say that in my capacity as a visitor I was careful not to err in this way.

It appeared to me that M. Foville was particularly observant of the general character, temperament, and configuration of his patients, and, although a very guarded phrenologist, had paid more than common attention to the relations of the skull to the developement of the principal divisions of the brain. His anatomical researches and views respecting the physiology and pathology of the cerebro-spinal system have lately been given to the public in a valuable Treatise, reviewed in your Twenty-fifth Number, and of which it is unnecessary for me to speak further. M. Foville has made curious and, I believe, original observations on the shape of the ear in different forms of insanity, and has noticed an analogy or resemblance between the developement of different portions of this organ and the brain of the patient. Of these views he was so obliging as to give me some explanation, illustrated by an extemporaneous diagram, and afterwards by corroborative examples. In some of the cases of dimentia, or of the lowest degree of intelligence, the flatness and defective form of the helix, anti-helix, and tragus, and the disproportionate enlargement and pendulosity of the lobe of the ear, and rounded clumsy shape of the outer edge of the auricle, were very striking. Subsequent observations have led me to believe these views to be exact as well as curious ; and they exemplify the abundance of eternal evidence available to the physician in relation to internal disorder. The diagnostics of Lavater fell into discredit by their idle and hasty application in the hands of his followers; but there are remarks in that great observer's works, even on so minute a matter as the natural growth and disposition of the *hair*, of the value of which I have only become conscious since my own observations have been accidentally directed to the hair as one of the external characteristics of melancholia, mania, and imbecility. Those familiar with the patients of any asylum can scarcely have failed to observe how frequently the disposition of the hair during the whole of a long maniacal paroxysm receives a peculiar modification; becomes raised, bristled up, *herissé*, and disordered beyond the control of comb or brush. The heavy masses of smooth hair in the melancholic must also have attracted attention. These characteristics are well portrayed, in some of the plates attached to Esquirol's work; and also in a spirited engraving entitled 'Narrenhaus.' It is not necessary to magnify the importance of these things; but they are not to be despised. They belong to the studies of which Celsus says, *adjuvant excitando artificis ingenium.* Not very long ago, M. Foville was called upon by an intelligent and philanthropic person, who appeared to take much interest in the management of lunatic asylums; and he was greatly struck with a conformation of ears in this gentleman which he had never previously observed, except in cases of mental irregularity or disorder. I happen myself to know that the individual who was the subject of this observation has had several attacks of insanity; and although now at large, and exhibiting considerable mental activity, has repeatedly been in confinement; circumstances of which M. Foville had no knowledge when he remarked what seemed to him to be an anomalous peculiarity.

I did not see at Charenton any cases of an actual disease of the external ear which seems to be especially if not exclusively allied with cerebral disorder, being perhaps only observed in the insane. It consists of general but not acute inflammation, and slow enlargement by thickening and effusion, of the whole of the outward ear; sometimes attended with much suffering; often becoming mitigated and then renewed, and never wholly removed, but not affecting the sense of hearing, except by mechanical obstruction of the meatus. There are always some examples of this affection at Hanwell, chiefly among the male subjects; but it cannot be clearly associated with any one original form of disease of the brain. It is allied in one case with epilepsy and weakness of the mental faculties; in others with general paralysis, and the incoherence consequent on mania; in others with confirmed dementia.

At Charenton, as in all large asylums, there are many examples of the form of paralysis, first fully described by M. Calmeil, and now generally known to the physicians of such institutions, although little recognized out of them, and called paralysie général. M. Foville does not appear to be at all more sanguine than myself with respect to the means of cure of this form of malady. Daily experience confirms my opinion that it is invariably connected with irremediable organic change in the membranes or substance of the brain, or with effusions too extensive to be removed.

There was more agitation in the wards devoted to the least tranquil of the female patients at Charenton than I had observed at the Salpêtrière; but no great violence of conduct or manner: one apartment only presented an unfavorable exception, an apartment belonging to the old portion of the buildings allotted to the most turbulent. In this room I was strongly reminded of what was once familiar to me at Hanwell. The room seemed on entering it to be filled with noisy voices. Four or five of the female patients were sitting in those heavy coercion-chairs which are now rarely to be met with; one was leaning back in the chair with an air of sullen depression, and quite silent, appearing, indeed, to be in a state of mere dementia. Two or three others were struggling and declaiming with great vehemence and loudness; and another was talking very volubly but with perfect good humour. I was in some degree prepared for this spectable by having just read in the 'Quarterly Review' that M. Foville was opposed to the non-restraint system; a subject on which I had not heard his opinion. But still it appeared to me almost as shocking as if I had never witnessed such a scene before, and had forgotten the dangerous ferocity of the daily occupants of the old coercion-chairs at Hanwell, and the wild careering of patients bound up in strait-waiscoats, and the shouts, oaths, threats, and curses which used to greet us every morning in the refractory wards, until the abolition of violent methods of repression caused such scenes to be unknown, and any approach to them rare and of short duration. As M. Foville did not make a single remark on these cases, or on the subject of restraints, I felt it incumbent upon me to maintain an equal reserve. I recollected that when he visited Hanwell in 1840, the great experiment of dispensing with restraints there, although previously made at Lincoln, was only in the first stages of its progress, and embarrassed by great opposition and difficulty. I thought it probable that he might have read the recent Report of the Commissioners in Lunacy, in which circumstances were alluded to, calculated without explanation to throw discredit on the non-restraint system, as practised even now at Hanwell. Otherwise to a physician of so much good sense, and apparently so unprejudiced, I think I could have shown that no inconveniences were avoided by the restraints imposed on these poor women, which could not have been better obviated by less objectionable means; whilst some inconveniences were incurred by the use of restraints, that might have been advantageously and altogether avoided.

It appeared, from such inquiries as I had the opportunity of making, that in each of these examples of the use of the restraint-chair, the reason assigned was precisely similar to what is always alleged where restraints of any kind are still habitually employed. One patient would tear her clothes if at liberty; one would roll about the floor; one would break the windows; one would undress herself. Such reasons may surely be pronounced to be most unsatisfactory; each of these habits admitting of no very difficult remedy without restraints; and the tumult, the discontent, the air of neglect, the bad smells, prevailing in this as in every room where restraints are often used, constituting evils far greater than those which it is employed to prevent. Almost when writing these observations I have before me a fine old man, formerly a soldier, and recently brought to Hanwell, as too many are brought, merely to die. Broken down in constitution by service in a bad climate, he became enfeebled in body and mind; but, as his poor wife informs us, he was cleanly in his habits, and able to move about until lately fastened down in bed for weeks in one of the houses in which restraints are yet used with impunity; since which time he has become unable to walk, is suffering from ulceration of the back, is unable to attend to cleanliness, and lies helpless on a bed from which not all our care will ever make him rise again. I naturally mention this case because I have just been grieved by the sight of it; but similar instances are continually presented to our attention.

As M. Battelle has been furnished, at his express request, with patterns and models of all our Hanwell devices for meeting every inconvenience without tying the arms and legs of our patients or shutting them up in chairs, I hope he will be able to convince the officers of the asylums over which his administration extends, of their efficacy as well as their simplicity; and that their own experience will soon satisfy them of their preferableness to violence in any form. They cannot but find, that if a patient tears her clothes, it is not impracticable to dress her in strong clothing, not easily torn. If bedding and blankets are destroyed, strong cases will protect them; which restraints do not. If a patient undress herself, the substitution of a small lock for buttons or strings, securing the dress at the neck or waist, and at the wrists, is readily accomplished and renders undressing impossible. Dresses of a peculiar form easily obviate the removal of blisters, dressing, &c., in cases requiring them, and remedy many inconvenient uncleanly habits. If some patients take delight in breaking windows, it is easy to guard such windows as are exposed to these accidents by wire-blinds of a light construction, between which and the windows plants may also be placed, and kept from damage. If they feel a wild gratification in rolling on the floor, it is easy to have a soft covering for the floor. If, more desperate, they would dash themselves against the walls, which they very rarely indeed think of doing, it is not difficult to pad and cushion the walls of their rooms. If they will not lie down, or if from epilepsy or weakness they fall out of bed, it is very easy to make the whole floor a bed, from which they cannot fall, or on which they can walk, without danger, until they prefer lying down. For all cases of extreme violence, seclusion, or free exercise with proper superintendence, are the only useful measures not strictly medical; if they fail to produce tranquillity, violent restraints will render it still more unattainable. There is no difficulty and no danger which may not be met by analogous contrivances, which have now been successfully used in several English asylums for some years. And the general results of these contrivances are, that the patients become calmer, more apt to receive the impressions of all humane treatment; that the attendants do not become nursed in and inured to inhumanity; and that the *health* of the patients, and their cure, become the first considerations. They enjoy the free exercise of their limbs; and yet, in the end, fewer windows are broken; fewer violences of all kinds are committed; the fierce excitement of the wards disappear; the life of the lunatic is rendered more tolerable, and that of the attendants and officers less anxious.

All my remarks have sufficiently shown that I think the general treatment of the patients in the French asylums is kind and judicious; but on recalling what I witnessed, I feel impressed with an opinion that the practice of resorting to restraint is not without its usual influence on the physicians who do so, although they may be somewhat unconscious of it. The practice tends to deaden the feelings to the actual condition of the patient,—an afflicted, suffering person; weak, wayward, and indolent, perhaps malicious or vicious, and yet innocent; above all helpless and unprotected, except by the physician and those whom he employs. In illustration of this, I will merely mention that th use of baths of all kinds, but especially in their severest forms, is professedly resorted to as much and as often for a punishment as for a remedy. That the shower-bath and the douche have a moral, as well as a physical effect on an insane patient is undeniable; and that both are, in certain cases and with certain precautions, useful, with both these intentions, may also be granted; but it is no less certain, that to resort to either of them solely as a moral remedy, or as a means of repression, is a practice fraught with peril to the humanity of the practitioner, and liable to cruel abuse in the hands of his attendants. So used, it may become an instrument of torture, not a remedy; and instead of being employed to cure irritation of the brain, may be applied to subdue and alarm the obstinate or the frantic, and even to forcea poor creature, whose energies are oppressed, to undertake the work only adapted to a healthy man. If the principle is once admitted that punishment and mortification are among the proper remedies for the insane, and that they ought to work, whether they work willingly or not, the austerity of cold and unfeeling officers will carry it to detestable excess. Combining it with the economy so important to all governing bodies which desire to conciliate the public, it becomes a pretext for lowering the diet, for neglecting the clothing, for withholding needful extras, except as the price of labor. For such offices, the effect of which is concealed, the officers of an institution may easily gain favour when really least deserving it. Certain it is, that the use of restraint seems, by some fatality, to be associated with innumerable negligences, of all which the patients are the victims. But much cold-hearted negligence, much severity even, may long and successfully prevail where restraints are ashamed to show themselves.

I must not, however, be unjust to Charenton, or to the asylums of Paris. When I recollect that in the Salpêtrière and the Bicêtre there are no resident physicians, the progress already made in abolishing restraints excites my surprise. Knowing the difficulties of the task, even to those constantly on the spot, I cannot sufficiently admire the courage, as well as the humanity of those who make the attempt in asylums in which they can only spend a few hours in a week, and to which the resources of their minds, and the suggestions of their kindness, can consequently have but transient application. and with faint results. In all the English and Scotch asylums in which restraint has been wholly abolished, there has been a resident medical officer zealous for its abolition; ready at any hour to make a prompt investigation into all the supposed obstacles to its success, and enabled to counteract opposing and sinister influences. I do not think its abolition has been effected, on a large scale, in any other circumstances.—*Brit. & For. Med. Rev.*

